# Effect of multifocal spectacle lenses on accommodative errors over time: Possible implications for myopia control

**DOI:** 10.1167/jov.23.3.3

**Published:** 2023-03-02

**Authors:** Saulius R. Varnas, Dinesh Kaphle, Katrina L. Schmid, Marwan Suheimat, David A. Atchison

**Affiliations:** 1 Carl Zeiss Vision Australia Holdings Limited, Adelaide, Australia; 2 Centre for Vision and Eye Research, Queensland University of Technology, Kelvin Grove, Australia; 3 Discipline of Optometry, Faculty of Health, University of Canberra, Bruce, Australia; 4 Centre for Vision and Eye Research, Queensland University of Technology, Kelvin Grove, Australia; 5 Centre for Vision and Eye Research, Queensland University of Technology, Kelvin Grove, Australia; 6 Centre for Vision and Eye Research, Queensland University of Technology, Kelvin Grove, Australia

**Keywords:** aberrometer, accommodative lag, autorefractor, myopia progression, progressive addition lenses

## Abstract

The study purpose was to improve understanding of how multifocal spectacle lenses affect accommodative errors and whether this changes over time. Fifty-two myopes aged 18 to 27 years were allocated randomly to one of two progressive addition lens (PAL) types with 1.50 D additions and different horizontal power gradients across the near-periphery boundary. Lags of accommodation were determined with a Grand Seiko WAM-5500 autorefractor and a COAS-HD aberrometer for several near distances with the distance correction and the near PAL correction. For the COAS-HD the neural sharpness (NS) metric was used. Measures were repeated at three-month intervals over 12 months. At the final visit, lags to booster addition powers of 0.25, 0.50, and 0.75 D were measured. Except at baseline, both PALs’ data were combined for analysis. For the Grand Seiko autorefractor, both PALs reduced accommodative lag at baseline compared with SVLs (*p* < 0.05 and *p* < 0.01 at all distances for PAL 1 and PAL 2, respectively). For the COAS-HD, at baseline PAL 1 reduced accommodative lag at all near distances (*p* < 0.02), but PAL 2 only at 40 cm (*p* < 0.02). Lags measured with COAS-HD were greater for shorter target distances with PALs. After 12 months’ wear, the PALs no longer reduced accommodative lags significantly, except at 40 cm distance, but 0.50 D and 0.75 D booster adds decreased the lags to those measured at baseline or less. In conclusion, for PALs to reduce accommodative lag effectively, addition power should be tailored to typical working distances and after the first year of wear should be boosted by at least 0.50 D to maintain efficacy.

## Introduction

Development and progression of myopia have affected human populations for a long time, but their impacts on society were limited while opportunities for good education were restricted. With the availability of universal education in developed countries and parents’ desire to ensure high-quality education for their children to secure well-paid jobs in the high added–value economies, these pressures are unlikely to ease any time soon.

Given the high rates of prevalence of myopia in many countries (Holden et al., 2016; Morgan et al., 2018), especially in big cities, and the known risks of myopia to eye health, it is imperative to find ways to at least slow down progression of myopia in primary school children, as well as students in secondary and tertiary educational institutions. With the establishment of the International Myopia Institute and some recent success in slowing myopia progression in randomized controlled clinical trials (Bao et al., 2022; Chamberlain et al., 2019; Lam et al., 2020; Walline et al., 2020; Yam et al., 2019), myopia management has come into the mainstream of optometric practice.

As well-designed and executed randomized clinical trials involving large number of subjects become available, meta-analysis can provide sufficient confidence in the efficacy of the specific treatments. A recent meta-analysis considered the efficacy of bifocal and multifocal spectacle lenses in slowing progression of myopia (Varnas, Gu, & Metcalfe, 2021). These lenses were found to be moderately effective in the first year, reducing progression by about 30% in children, but the efficacy tended to weaken over longer treatment periods. Evidence from a recent clinical trial of multifocal soft contact lenses suggests that higher addition powers improve efficacy of treatment (Walline et al., 2020).

When trying to address the issues with the efficacy of multifocal spectacle lenses applied to myopia control, we have adopted a hypothesis that it is the larger lag of accommodation in myopes than in emmetropes (Kaphle et al., 2022) that is driving the axial elongation of the myopic eye behind the fovea, which is the main cause of progressing myopia. For example, a possible mechanism for the decrease in myopia control effect of multifocal lenses over time may be a change in the accommodation system over time (i.e., an adaption to the near addition). Also, it is of considerable interest to understand how multifocal lenses modulate accommodative errors. A recent study has found that conflicting stimulation of peripheral retina to fovea during accommodation can affect accommodative responses (Labhishetty, Cholewiak, & Banks, 2019). Because multifocal spectacle lenses can have different variations of the power profile in the periphery, different lens designs could be capable of producing varying accommodation even when the foveal stimulus is the same. Schilling, Ohlendorf, Varnas, and Wahl (2017) measured accommodative responses, for a range of object distances using the Grand Seiko WAM-5500 autorefractor (Grand Seiko Co. Ltd., Hiroshima, Japan), in young myopic adults looking through the center of the near zone of four progressive addition spectacle lens (PAL) types having an addition power of 1.50 D and varying peripheral power designs. Lags of accommodation at 25 cm could not be contained within the limits of the depth of focus (DOF) for three of the PAL types. A recent pilot study of typical distances when children were viewing hand-held small electronic devices (Xu, Jaskulski, Bradley, Kollbaum, & Krueger, 2020) found that the mean viewing distance was 24 cm, and this decreased to 21 cm when viewing small text (8 point). This indicates that the 1.50 D multifocal lens is unlikely to reduce the accommodative lag adequately to ensure no, or only low, hyperopic defocus for near vision during such activities.

In a previous article (Kaphle et al., 2022) we confirmed earlier reports (Labhishetty, Cholewiak, Roorda, & Banks, 2021) that Grand Seiko autorefractors tend to overpredict accommodative lags relative to refractions derived from aberrometer measurements for natural pupils and suitable metrics incorporating higher order aberration components of refraction.

The aim of this study was to improve the understanding of how progressive addition lenses affect accommodative errors and their changes over time, so that more effective prescribing strategies can be developed for the use of these accommodative aids to slow progression of myopia. We compared Grand Seiko WAM-5500 autorefractor and COAS-HD aberrometer measurements in young adult myopes to evaluate the reduction of accommodative lag when wearing two different PAL designs having a 1.50 D addition power. We examined how the lags changed over a 12-month clinical trial. At the last visit we measured accommodative responses to a range of booster addition powers. Although the main instrument was the COAS-HD for the reasons given above, the Grand Seiko instrument was included so we could compare baseline measurements with an earlier study of Schilling et al. (2017) considering different PAL designs.

## Methods

### Participants

This study adhered to the tenets of the Declaration of Helsinki. The Queensland University of Technology Human Research Ethics Committee approved the protocol. All participants signed the informed consent form before enrolment into the study. A double-masked two-arm parallel randomized controlled trial was conducted over 12 months of follow-up at the School of Optometry and Vision Science. This trial was registered with the Australian New Zealand Clinical Trials Registry (Reg. No. ACTRN12619000538145).

Fifty-two myopes aged between 18 and 27 years were recruited from Queensland University of Technology students and their acquaintances. They were the myopic participants of the Kaphle et al. (2022) study. Young adults have been used in preference to children because of the difficulties of maintaining attention of children during the lengthy and tedious measurement process. They had non-cycloplegic SER between −0.75 D and −6.00 D. All participants had good ocular and general health, anisometropia and cylinders < 1.75 D, and no past or current history of myopia control treatment. Participants were allocated randomly one of two PAL designs, both of which had an addition power of 1.50 D at the near reference point (NRP).

A questionnaire was administered at each visit to assess compliance of new spectacles. If there were any issues with adaptation, they were resolved by troubleshooting. Compliance issues, if any, were recorded. The proportion for participants wearing spectacles at least 12 hours or more ranged from 90% to 95% for all follow-up visits. There was no significant difference between the two designs regarding compliance or number of hours of wear.

**Figure 1.**
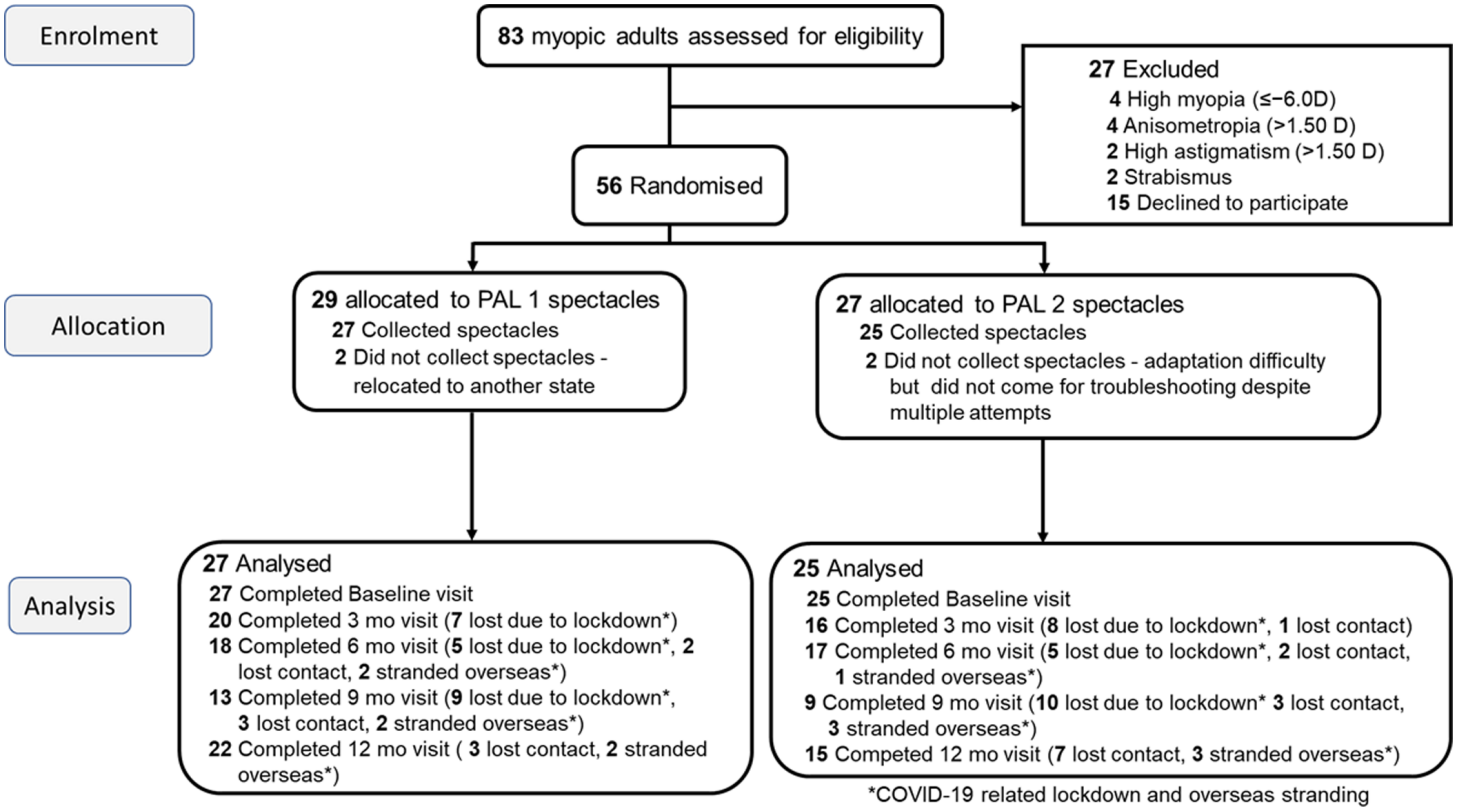
Flow chart of the trial.

Figure 1 shows the flow chart of the 12-month trial. The trial was affected by the COVID-19 pandemic, which contributed to a considerable amount of missing data when participants could not return for scheduled visits.

#### Lenses

**Figure 2.**
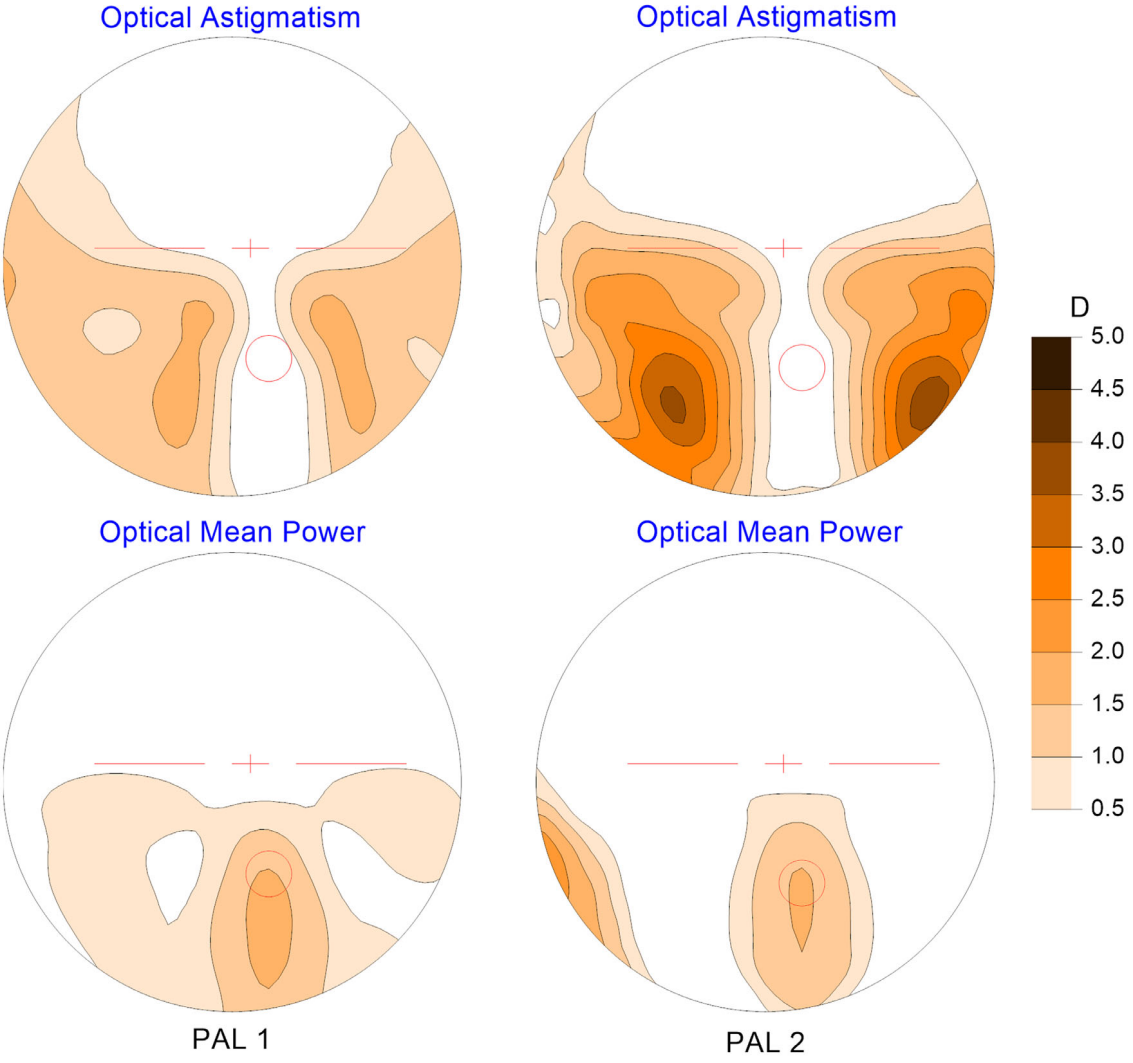
Distribution of ray traced astigmatism and relative mean power offset to zero at the distance reference point for the PAL designs used in the trial. They represent the optics of these lenses for a typical −2.50 D distance power prescription over the diameter of 50 mm. The location of the fitting cross and the center of the near reference point as a 2.5 mm radius circle are marked as *red color ink marks*.

PAL 1 was a design with low peripheral mean power gradients having a corridor length from the Fitting Cross (FC) to the NRP equal to 14 mm, whereas PAL 2 was a design with high peripheral mean power gradients and relatively narrow near zone with the corridor length of 12 mm (Figure 2). PAL 2 had a high peripheral astigmatism, which meant that there was a substantial difference between the sagittal and tangential power gradients in the horizontal direction away from the NRP of this PAL. The PAL 1 and PAL 2 designs were similar to, but not the same as PAL 1 and PAL 4, respectively used by Schilling et al. (2017). Both lens designs were manufactured by free-form technology, with spherical front surfaces and back surfaces individually optimized for a prescribed Rx and calculated as a combination of the progressive surface and any prescription cylinder.

Block randomization (block size of 10) was implemented by an unmasked investigator (S.R.V.) using an online resource (http://www.randomization.com/). Both the examiner (D.K.) and participants were masked regarding the lens design allocations. Three lenses were manufactured for each participant. A pair of left and right eye lenses was fitted into a regular frame chosen by a participant from a range of frames, subject to the minimum fitting height requirement of at least 2 mm clearance below the NRP to the frame edge. The third lens was made for the dominant eye and fitted into a ring to be inserted into a trial frame during refraction measurements, and was centered at the NRP. There was an axis mark at the edge of the lens so that when the mark was aligned at the 180° axis, it would correspond to the participant's Rx axis. The position of the trial lens within the trial frame was adjusted so that participants looked through the NRP at the center of the lens for near testing distances. Accommodations measures were performed monocularly during monocular viewing.

After baseline measurements, in which subjects’ accommodative responses were measured with single vision distance lenses (SVLs) and PALs, there were four follow-up visits after every three months of wearing PALs on a full-time basis. Measurements of refractive state when wearing the distance prescription and viewing the target at either 4 or 5 m distance were repeated every six months, whereas refractions when looking at the close distances through the near zone of the PAL were repeated every three months. At the final 12-month follow-up, the accommodative responses to the near distances were recorded when the 1.50 D addition of the PAL was boosted by three increments: 0.25, 0.50, and 0.75 D. The boosters were implemented as spherical power trial lenses inserted into the trial frame over the PAL trial lenses.

### Measurements

Eye and vision testing included ocular health assessment, slit-lamp biomicroscopy, intraocular pressure measurement, direct ophthalmoscopy, automated refraction (Grand Seiko Auto Ref/ Keratometer WAM-5500), and subjective refraction. Best-corrected Snellen visual acuity was 6/6 or better in all participants. Full distance refractions were provided by lenses in a trial frame. Subjective amplitude of accommodation, heterophoria, AC/A and CA/C ratios were measured as described by Kaphle et al. (2022).

### Accommodative response: Grand Seiko autorefractor

Full descriptions of refraction measurements appear in Kaphle et al. (2022), and so only modified descriptions, including variations, are given here.

**Figure 3.**
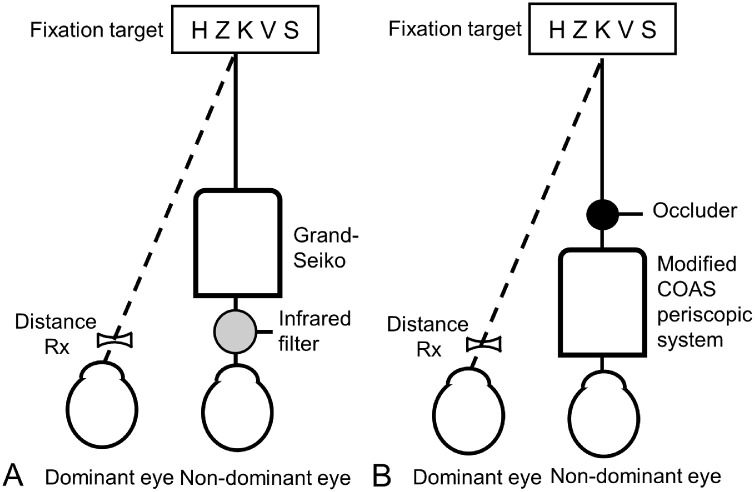
Experimental setup for measuring accommodative response with (a) the Grand Seiko autorefractor (GS) and (b) the COAS-HD aberrometer. Not to scale. Background target luminance was approximately 100 cd/m^2^. Participants were instructed to focus on letters and keep them “as clear as possible” during measurement. For GS alignment, room lights were dimmed so that targets appeared at the center of the instrument's red alignment ring, and the participant's non-dominant eye was measured along the axis of the instrument by translating, as necessary, the external letter target to align at the center of the instrument's ring. For COAS-HD alignment, the non-dominant eye was measured along the axis of the instrument by translating, as necessary, the external letter target to align with the super luminescent diode source of the instrument faintly seen by the non-dominant eye. See text for other details.

Accommodative response was measured with the Grand Seiko autorefractor WAM-5500 (Grand Seiko Co. Ltd.). The dominant eye was determined by a sighting alignment (pointing-a-finger) test (Seijas et al., 2007). The dominant eye viewed the target and drove the accommodation response, while the refraction of the other eye (i.e., the consensual response) was measured. This was achieved by placing a distance SVL lens, a specially fitted PAL trial lens, or a combination of the PAL trial lens and a booster add power trial lens correction in front of the dominant eye, while an infrared pass filter (87C; Kodak Corp., Rochester, NY, USA) in front of the nondominant eye occluded its view but transmitted the instrument's infrared radiation (Figure 3a). The lenses were decentered according to inter-pupillary distance, vertex distance, and target distance. For distance the fixation target was a cross presented at 4 m, and at each of 40, 33, 25, and 20 cm five high-contrast letters were presented in a row, each subtending 12.5 min of arc vertically (6/15 letter size). The participants were instructed to focus on the letters and keep them “as clear as possible” during measurements. Five measurements were taken for each testing distance in a decreasing order of distance. Average spherical equivalent refraction and *J*_0_ and *J*_45_ astigmatism were calculated using power vector analysis (Thibos, Wheeler, & Horner, 1997).

Accommodation lags (LoA) were calculated as described by Kaphle et al. (2022), but considering the modification of the prescription due to the addition power of the lens when looking through the NRP:

$$\begin{array}{ccc} & & \mathrm{AS}=\frac{\mathrm{Rx}}{1-\mathrm{VD}\;\times \;\mathrm{Rx}}\\ & & \;-\frac{1+\left(\mathrm{TD}+\mathrm{VD}\right)\;\left(\mathrm{Rx}+\mathrm{ADD}\right)}{\mathrm{TD}-\mathrm{VD}\left(\mathrm{TD}+\mathrm{VD}\right)\;\left(\mathrm{Rx}+\mathrm{ADD}\right)} \end{array}$$

$$\begin{array}{ccc} & & \mathrm{AR}=\left[\mathrm{Grand}\;\mathrm{Seiko}\;\left(4\;m\right)+0.25\right]\\ & & \;-\mathrm{Grand}\;\mathrm{Seiko}\;\left(Near\right) \end{array}$$

LoA=AS-AR

Here AS is the accommodation stimulus, AR is the accommodation response, Rx is the spherical equivalent power of the SVL worn by the dominant eye and is equal to the subjective refraction of that eye at the spectacle plane, ADD is the addition power of the PAL when looking through the NRP, VD is distance from the spectacle plane to the cornea and is always positive, TD is (negative) near testing distance from the eye, Grand Seiko (4 m) is the Grand Seiko measured SER at the corneal plane for a 4 m target, Grand Seiko (Near) is the Grand Seiko measured SER at the corneal plane at one of the four near testing distances, and the sign of LoA is positive for an accommodative lag. Target distances were modified slightly to consider the adduction required for the dominant eye (Kaphle et al., 2022). In the equations, LoA, AS, AR, ADD, Grand Seiko (*4 m*) and Grand Seiko (*Near*) are in diopters (D), and TD and VD are in meters (m).

### Accommodation response: COAS-HD aberrometer

The Complete Ophthalmic Analysis System (COAS-HD) Hartmann–Shack Aberrometer (WaveFront Sciences, Albuquerque, NM, USA) was modified by adding a periscopic system between the eyes and the COAS-HD that enabled placing the eyes above the level of the instrument while still measuring the refraction through the periscopic system (Mathur & Atchison, 2013). The internal fixation target of the COAS-HD was turned off. Like the Grand Seiko, the dominant eye drove the response, and the consensual response of the nondominant eye was measured along the instrument axis. This was achieved by placing a trial lens in front of the dominant eye and by placing an occluder on the line between the first (hot) mirror of the periscopic system and the letters so that the target was not visible to the nondominant eye (Figure 3b). The vertical dimension does not show on the figure.

Both distant (5 m) and near targets consisted of 5 high contrast, 6/15 sized letters in a row on a tablet or smart phone. As for the Grand Seiko autorefractor, the participants were asked to focus on the letters and keep them “as clear as possible" during the measurement. Measurements were conducted in a decreasing order of distance. Four measurements were taken for each distance, and results were averaged, after any obvious outliers were removed.

Aberration data were exported as Zernike coefficients up to sixth order for 550 nm wavelength. Objective refractions from the aberrometer measurements for natural pupil diameters, taking into account aberrations up to 6^th^ order and maximizing visual quality by using the neural sharpness (NS) metric, were computed using a MATLAB (MathWorks, Natick, MA, USA) program developed at the University of Indiana and described by Thibos, Hong, Bradley, and Applegate (2004). Accommodative stimulus, accommodative response (relative to the response for a distance target), and accommodative lag were calculated as for the Grand Seiko, except that the adjustment constant to calculate the response for infinity was 0.2 D rather than 0.25 D (as target distance was 5 m instead of 4 m for the Grand Seiko).

### Analysis

Previously we concluded that, of five different methods consisting of GS measurements and four methods with the COAS-HD, the COAS-HD measurement using the neural sharpness (NS) metric with natural pupil sizes was the most reliable and representative for prediction of accommodative lag differences between emmetropes and myopes (Kaphle et al., 2022). Accordingly, only this method was considered for comparisons involving the COAS-HD aberrometer.

Lags were measured at 40, 33, 25 and 20 cm for the Grand Seiko autorefractor, and at 40, 33 and 25 cm for the COAS-HD aberrometer. The target distances were converted to accommodative stimuli in diopters. Since the accommodative stimuli are dependent on the power of the lenses worn and their back vertex distances, this conversion for the corrected myopes resulted in a range of dioptric stimuli for each target distance, and hence results are discussed in terms of target distance.

Statistical analysis was performed in R (V3.5.2) software. Exploratory data analysis revealed few outliers, indicative of normality of the distributions. Therefore Student's *t*-tests were performed. Differences in accommodative lags when wearing different lenses were compared at baseline using paired *t*-tests when the two conditions being compared were measured on the same subject (e.g., SVL to PAL comparison) and unpaired *t*-tests when the means of the cohorts wearing different PAL lenses were compared. A one-tailed Student's *t*-test with an appropriately formulated alternative hypothesis (lag greater for single vision than for a PAL) was used for the paired comparisons, and two-tailed tests were used for the unpaired comparisons between the two cohorts.

For the lens trial, the variable being analyzed was the difference in accommodative lags between the baseline visit when wearing an SVL and follow-up visits when wearing a PAL. Only one measure of refraction was used in this analysis—that derived from the COAS-HD aberrometer with the NS metric for the natural pupil size. To maximize the power of the statistical inferences, we tested pairwise comparisons between the lags at the follow-up visits and the baseline. Because participants attending the follow-up visits were not always the same, we selected the compared cohort at the baseline visit to include only those participants that completed that follow-up visit. This resulted in partially overlapping but somewhat different cohorts being compared at different follow-ups.

## Results

### Comparison between groups

Table 1 compares the two PAL wearing groups for the factors that might influence accommodative response. The values were obtained according to the methods described by Kaphle et al. (2022). There were no significant differences between the groups.

**Table 1.** Baseline demographic and ocular characteristics of myopes. Data are mean (standard deviation). Comparison between groups made using unpaired t-tests, *except chi-squared test for gender, race and family history of myopia. East Asian comprised 18 Chinese, 1 Japanese, 3 Vietnamese, 3 Indonesians, and 1 Filipino. South Asians comprised 10 Nepalese, 9 Indians and 1 Sri Lankan.

Characteristic	PAL 1 (*n* 27)	PAL 2 (*n* 25)	Unpaired *t*-test, p^*^
Age, y	22.4 (3.4)	21.6 (2.3)	0.32
Gender, female	17 (63.0%)	14 (56.0%)	0.61
Race			0.68
Caucasian	4 (14.8%)	2 (8.0%)	
East Asian	13 (48.2%)	13 (52.0%)	
South Asian	10 (37.0%)	10 (40.0%)	
Last change in Rx, months	17.0 (15.3)	14.3 (11.7)	0.50
Refractive error, D (mean both eyes)			
** **SER	−2.59 (1.61)	−2.31 (1.53)	0.53
** ** *J*_0_	0.15 (0.29)	0.27 (0.33)	0.23
** ** *J*_45_	0.02 (0.07)	−0.02 (0.11)	0.09
Axial length, mm	24.61 (0.96)	24.55 (1.16)	0.84
Amplitude of accommodation, D	8.32 (0.88)	8.71 (1.02)	0.18
Near heterophoria, Δ (− is exo)	−2.4 (6.7)	−3.3 (4.7)	0.56
Distance heterophoria, Δ	−0.8 (3.7)	−0.5 (2.2)	0.71
AC/A ratio, Δ/D	2.7 (1.0)	2.4 (0.9)	0.28
CA/C ratio, D/Δ	0.036 (0.018)	0.042 (0.027)	0.36
Family history of myopia, n (%)			0.38
Neither parent	5 (18.5)	3 (12.0)	
** **One parent	17 (63.0)	13 (52.0)	
** **Both parents	5 (18.5)	9 (36.0)	

### Baseline measures

**Figure 4.**
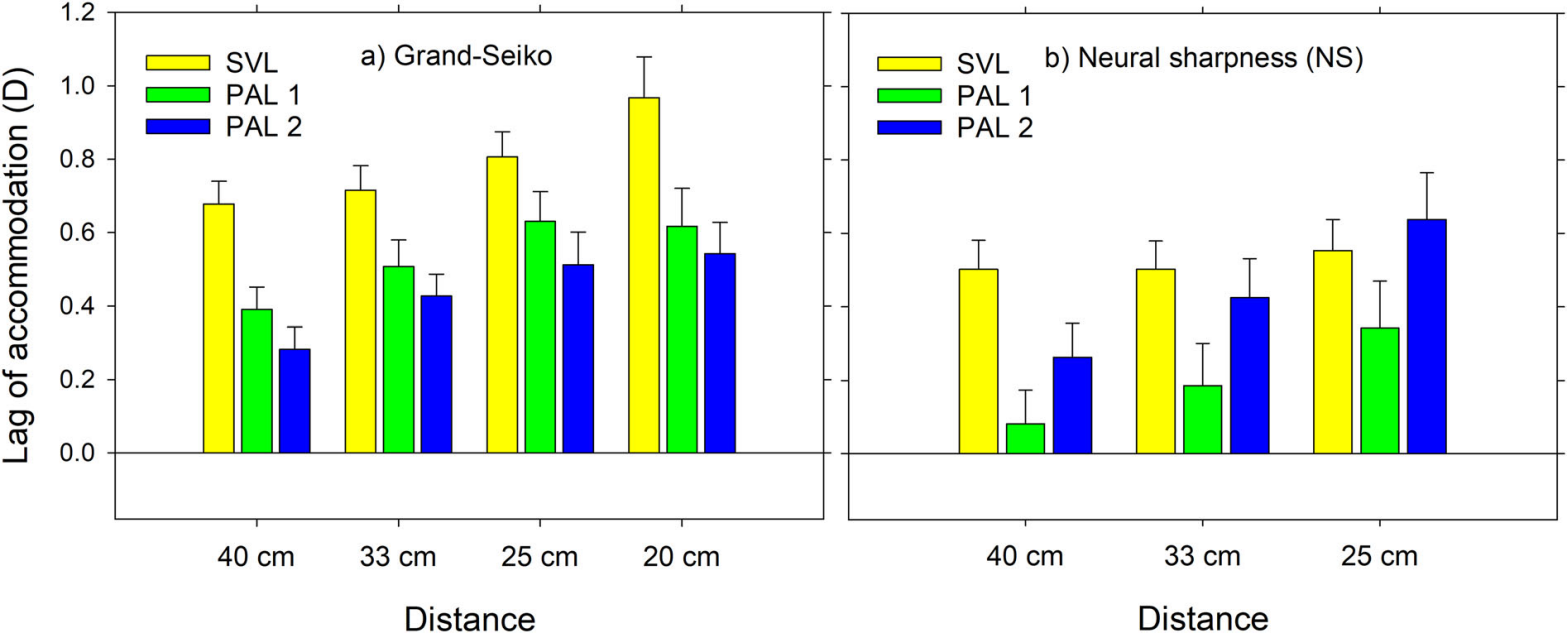
Mean lags of accommodation at baseline (a) with the Grand Seiko autorefractor and (b) derived from COAS-HD measurements using the NS metric. Results are for single vision distance lenses and for two PAL types with a 1.50 D addition, and at four and three viewing distances for the Grand Seiko and NS, respectively. *Error bars*: standard errors.

Figure 4 shows mean estimates of accommodative lags at the baseline visit using the Grand Seiko autorefractor and the COAS-HD with the NS metric. Overall accommodative lags were lower for the NS metric than for the Grand Seiko, with the differences being 0.2 to 0.25 D for SVL and 0.3 D for PAL 1, but with little change for PAL 2. Because lags were similar for the two PALs with the Grand Seiko, this suggests an effect of PAL design of periphery on accommodative responses only detectable with the COAS aberrometer. Use of the NS metric showed higher accommodative lags with decrease in target distance for PALs, but little change for the SVL.

Statistical inference test results for the lags of accommodation for Grand Seiko autorefractor measurements are displayed in Tables 2 and 3, and the corresponding results for the COAS aberrometer with the NS metric are shown in Tables 4 and 5. We used one-sided alternative hypotheses for paired *t*-tests as follows: (1) the accommodative lag will be greater wearing SVL than when wearing either of the PALs; (2) The lag will be greater for higher accommodative stimuli. To compare PAL 1 and PAL 2, where different cohorts of subjects were compared and there was no clear expectation which of the two PALs was likely to cause larger lags, we used a two-sided alternate hypothesis with an unpaired *t*-test.

**Table 2.** Results of hypothesis testing for the point estimates of the mean accommodative lags from the Grand Seiko autorefractor under different lens wearing conditions when the target distance was the same. The values in the second, fourth and sixth columns are the mean lag differences between lens conditions (first condition – second condition).

Comparison	40 cm	*p* value	33 cm	*p* value	25 cm	*p* value
SVL vs PAL1	0.284	**0.003**	0.216	**0.042**	0.172	**0.046**
SVL vs PAL2	0.405	**<0.001**	0.277	**0.001**	0.277	**0.009**
PAL1 vs PAL2	0.104	0.232	0.085	0.369	0.126	0.299

**Table 3.** Results of the hypothesis testing for the point estimates of the mean accommodative lags from the Grand Seiko autorefractor between different target distances when the lens worn was the same. Other details are as for Table 2.

Comparison	SVL	*p* value	PAL1	*p* value	PAL2	*p* value
25cm vs 40cm	0.115	**0.019**	0.244	**<0.001**	0.223	**<0.001**
25cm vs 33cm	0.079	0.065	0.121	**0.010**	0.08	0.092
33cm vs 40cm	0.036	0.222	0.123	**<0.001**	0.142	**0.001**

**Table 4.** Results of the hypothesis testing for the point estimates of the mean accommodative lags from the COAS-HD aberrometer/NS metric under different lens wearing conditions when the target distance was the same. Other details are as for Table 2.

Comparison	40 cm	*p* value	33 cm	*p* value	25 cm	*p* value
SVL vs PAL1	0.419	**<0.001**	0.356	**<0.001**	0.250	**0.016**
SVL vs PAL2	0.249	**0.013**	0.033	0.365	−0.150	0.877
PAL1 vs PAL2	−0.182	0.173	−0.232	0.142	−0.296	0.107

**Table 5.** Results of the hypothesis testing for the point estimates of the mean accommodative lags from the COAS-HD aberrometer/NS metric between different target distances when the lens worn was the same. Other details are as for Table 2.

Comparison	SVL	*p* value	PAL1	*p* value	PAL2	*p* value
25 cm vs 40 cm	0.060	0.180	0.283	**<0.001**	0.375	**<0.001**
25 cm vs 33 cm	0.050	0.160	0.142	**0.021**	0.213	**0.003**
33 cm vs 40 cm	0.003	0.478	0.105	0.072	0.162	**0.008**

For the Grand Seiko autorefractor, both PAL 1 and PAL 2 reduced the accommodative lag significantly compared with the single vision distance lenses, with the PAL 2 showing a greater effect although the difference between PAL 1 and PAL 2 efficacy was not significant (Table 2). Table 3 shows significance only between the lags for the 25 and 40 cm distances when wearing an SVL, but there were more significances for the PALs and in particular for PAL 1 where all comparisons give statistically significant results.

The statistical inferences based on the COAS-HD with the NS metric are somewhat different from those of the GS measurements. For NS, PAL 1 reduced accommodative lag significantly compared with SVL at all distances, but PAL 2 did so only at 40 cm (Table 4). Although the difference in lags between PAL 1 and PAL 2 had the opposite sign to that derived from GS, it was still not significant (*p* > 0.1 at all viewing distances). Table 5 reveals that variations in target distance over the range between 40 and 25 cm did not produce significant variation in accommodative lags when a single vision lens was worn, but there was an increase in lag when wearing the PALs with the effect of PAL 2 being the more pronounced (*p* < 0.01 in all its comparisons).

### Temporal changes of accommodative lags

The trial was affected by the COVID-19 pandemic, which contributed to approximately 30% dropout rates at the three-, six- and 12-month follow-ups, while the nine month follow-up had a 60% dropout rate with only 22 out of 52 participants completing it. The original intention of the randomized clinical trial was to compare the effect of two interventions (PAL designs) having the same addition power in the center but different horizontal gradients of power in the periphery on the maintenance of the reduction of accommodative lag compared to the single vision lenses. As it turned out, the biggest differences in the reduction of accommodative lag between the two interventions were observed at baseline, but they were still not statistically significant. Considering this, we analyzed the temporal changes in accommodation of the combined group of PAL 1 and PAL 2 wearers.

**Figure 5.**
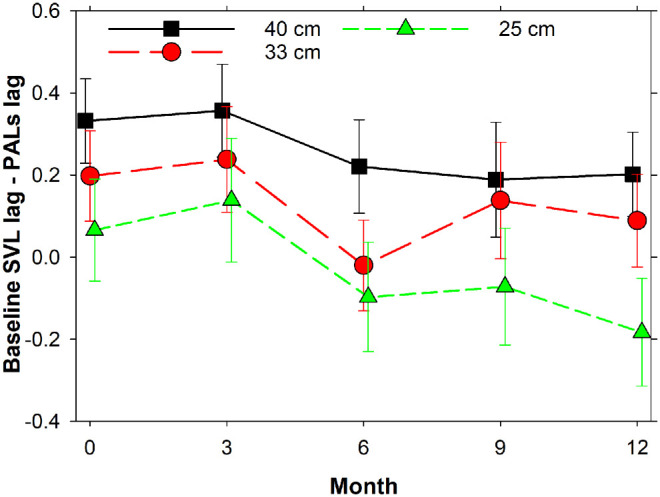
Accommodative lag of the single vision lens at baseline relative to the time-dependent lag of either PAL 1 or PAL 2. The Neural Sharpness metric with the COAS-HD was used for the analysis. *Error bars*: standard errors. As explained in the text, different cohorts were tested at three, six, nine, and 12 months.

Figure 5 shows the accommodative lag of the single vision lens at baseline relative to the time-dependent lag of either PAL 1 or PAL 2 using the Neural Sharpness metric with the COAS-HD. For participants who completed the 12-month follow-up, at baseline the mean reductions of accommodative lag with the PALs relative to SVLs were 0.35 D (*p* < 0.001), 0.23 D (*p* = 0.003), and 0.09 D (*p* = 0.11) for 40, 33, and 25 cm target distances, respectively, results which are similar to the baseline results shown in the figure for the whole group. The temporal variation of accommodative lags indicates a general trend of diminishing effect of the intervention over time. Most of the change appears to occur between the three- and six-month visits. At the 12-month follow-up, PALs still reduced the accommodative lag, compared with the SVLs at baseline, at 40 cm (*p* = 0.010) but not for shorter distances (*p* > 0.15).

### Effects of booster additions

**Figure 6.**
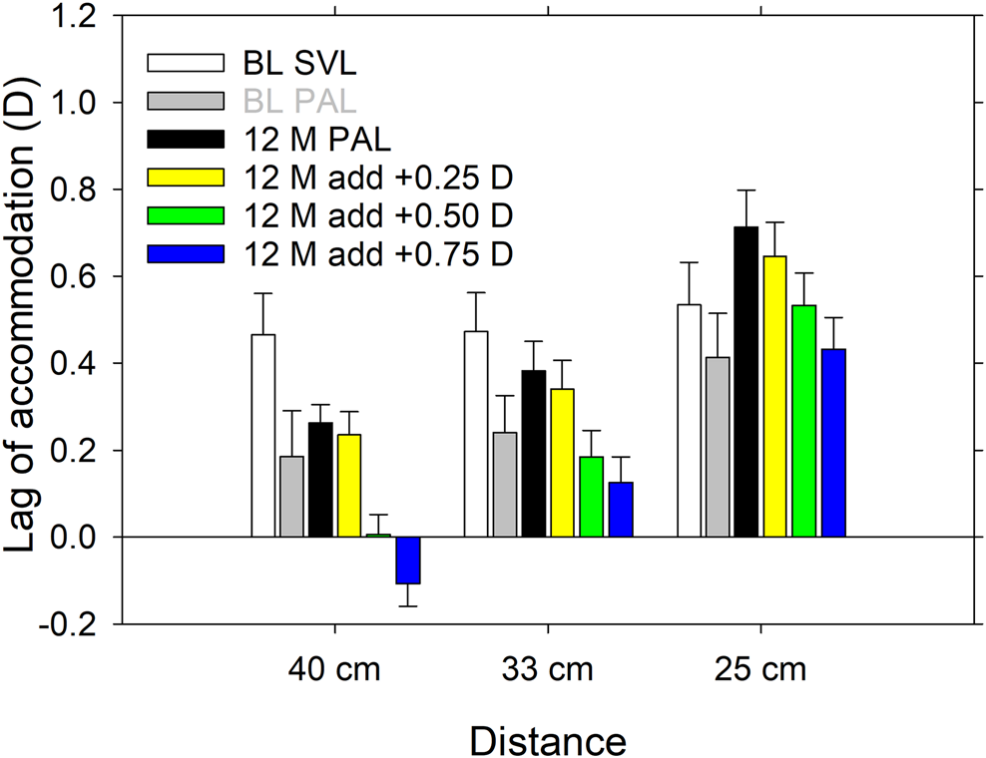
Comparison of lags of accommodation in the subset of wearers who completed the 12-month follow-up, at baseline when wearing SVLs and PALs, and when wearing PALs with a 1.50 D addition and three different booster addition powers at the 12-month follow-up. The Neural Sharpness metric with the COAS-HD was used for the analysis. *Error bars*: standard errors.

At the 12-month follow-up, accommodative responses were also measured when the addition powers of the PALs were boosted by 0.25, 0.50, and 0.75 D, respectively (i.e., when participants were looking through 1.75, 2.00, and 2.25 D additions). Figure 6 shows the mean lags of accommodation at the baseline visit when wearing both SVLs and PALs, as well as the corresponding mean values in the same cohort after the 12-month follow-up with the PALs and the booster additions. The Neural Sharpness metric with the COAS-HD was used. Due to the large loss to follow-up (Figure 1), the PAL groups were again combined in the analysis. The booster adds tended to reduce the accommodative lag. The booster adds of 0.50 D were effective in reducing the lag to below the depth-of-focus, which is similar to lags occurring in emmetropia (Kaphle et al., 2022), at 40 cm and 33 cm but not for 25 cm.

We ran Student's *t*-tests to compare lags of accommodation with PALs at baseline and the 12-month follow-up with different booster additions using one-sided paired comparisons with an alternative hypothesis that the lag at baseline is lower than after the 12-month follow-up. The 0.25 D booster made no statistically significant change at any target distance, and the lag was still higher than baseline for 25 cm (*p* = 0.03). The 0.50 D booster was sufficient to reduce the lag to at least the baseline level or lower at all target distances (*p* > 0.1). The 0.75 D booster lowered the accommodative lag further, with a lead of accommodation measured at 40 cm distance (*p* > 0.4).

## Discussion

### General

The aim of this study was to improve our understanding of how PALs affect accommodative errors and their changes over time. At baseline with the Grand Seiko autorefractor, both PALs reduced accommodative lag compared with that obtained with single vision lenses (SVL), with mean effects of 0.22 D and 0.32 D for PAL 1 and PAL 2, respectively. Lags for the PALs increased for higher accommodative stimuli (shorter target distances). At baseline with the COAS-HD, PAL 1 reduced accommodative lag compared with that obtained with SVLs for all stimuli, but PAL 2 showed a significant effect only at the smallest stimulus of 2.5 D. As for the Grand Seiko, lag for the PALs increased for higher accommodative stimuli. The reduction in lag at baseline for the PALs compared with the SVL (mean 0.22 D) weakened over time and was only significant after 12 months for the largest near target distance (40 cm). At the final visit, 0.50 D and 0.75 D booster adds were successful in restoring mean lags of accommodation to baseline levels or lower.

### Comparison with Schilling et al.

**Figure 7.**
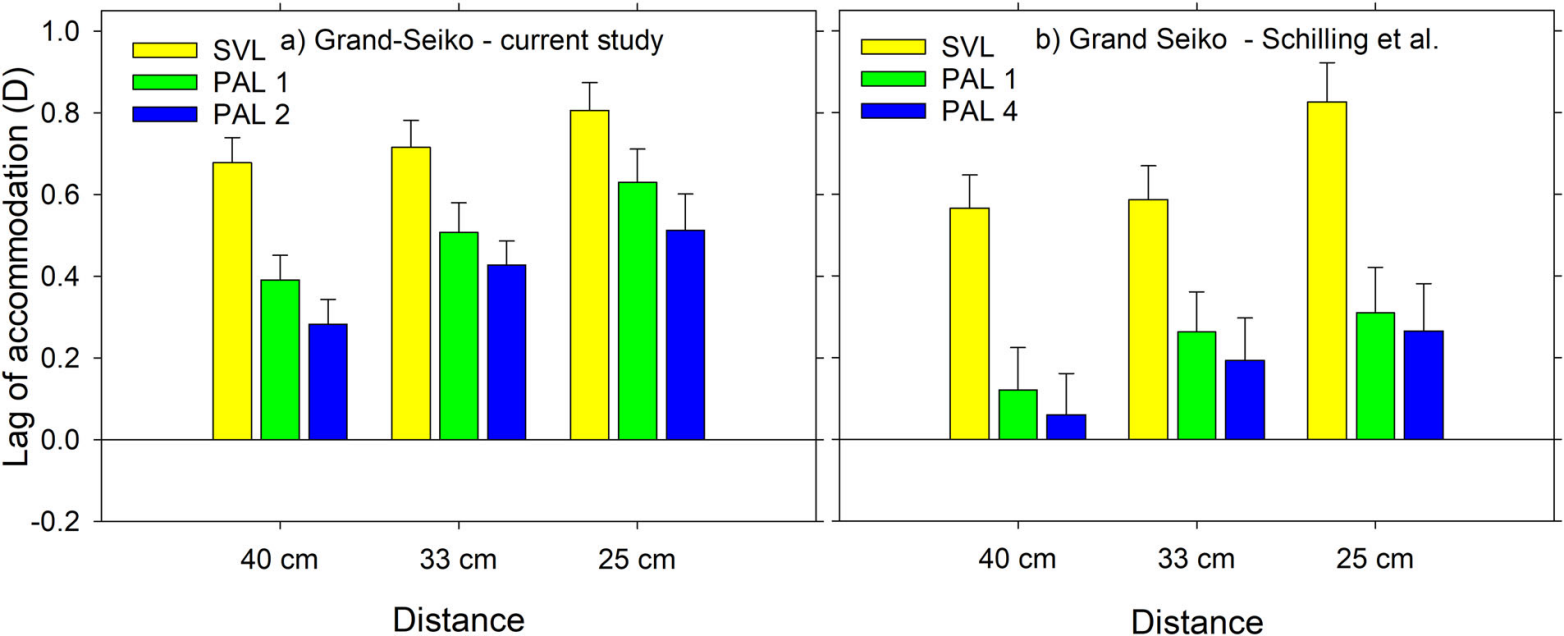
Mean lags of accommodation with the Grand Seiko autorefractor for a) the current study and b) the study of Schilling et al. (2017). Results are for single vision distance lenses and for two PAL types with a 1.50 D addition, and three viewing distances. PAL 1 and PAL 4 in the Schilling study are similar to PAL 1 and PAL 2, respectively, in the current study. The results in a) are those for Figure 3a except that the 20 cm data have been removed. Results in b) have been recalculated with the accommodative stimulus adjusted for oblique target distance. *Error bars*: standard errors.

The closest study, using similar methodology and similar lens designs, is that of Schilling et al. (2017) using the Grand Seiko. Figure 7 shows our Grand Seiko results at the baseline visit and Schilling et al.’s results, with the latter adjusted to account for the oblique viewing of the dominant eye. As in our study, Schilling et al. found that PALs reduce accommodative lags and that lags were higher as target distance decreased. Our results showed slightly higher accommodative lags than Schilling et al. with the SVL at the 40 cm and 33 cm distances (by ≈0.1 D), and in very close agreement at the 25 cm distance (within 0.02 D). With 1.50 D addition PALs, our participants had smaller accommodative lags than with the SVL condition, but the reductions were smaller than in the Schilling et al. study by 0.1 to 0.3 D when similar lenses are compared (PAL 1 in this study with PAL 1 in Schilling et al.’s study, and PAL 2 in this study with PAL 4 in Schilling et al.’s study).

**Figure 8.**
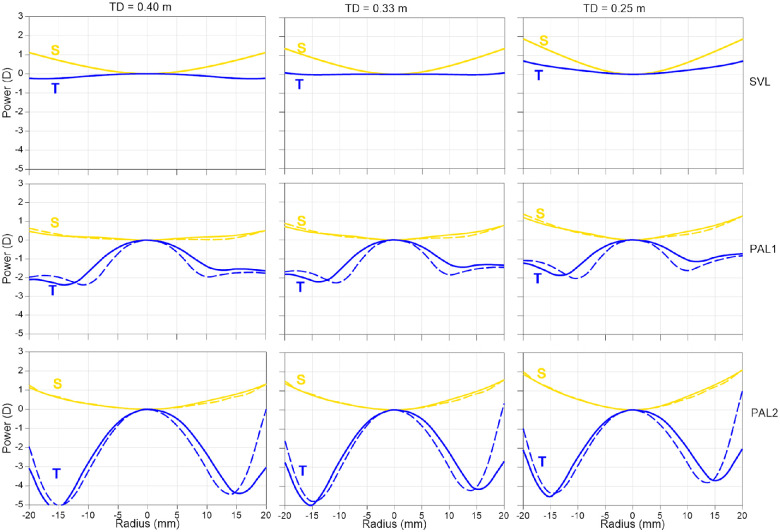
Theoretical sagittal (*yellow lines*) and tangential (*blue lines*) relative power profiles ray traced for the static myopic eye having the − 2.50 DS distance correction looking at 40, 33, and 25 cm distances when wearing lenses. Radial scan coordinates for ray tracing are on the front surface of the lens. Negative values for radius correspond to the temporal sides of lenses. It is assumed that the eye is perfectly accommodating for each distance for central vision. The top line shows a single vision spherical trial lens of −2.50 D having the base curve of 2.79 D (in 1.53 index), the second line shows the PAL 1 design used in this study (*solid plots*) and the PAL 1 design from Schilling et al. (*dashed plots*), and the bottom line shows the PAL 2 design in this study (*solid lines*) and the PAL 4 design from Schilling et al. (*dotted line*). The PALs have 2.40 D spherical base curves, and progressive designs are on the back surface of the lenses. The power profiles of lenses are different for different target distances because the obliquity of rays intersecting the lens surfaces depend on the target distance.

The small differences in results between the two studies may be in part due to peripheral design differences in our PAL lenses and those used by Schilling et al. This was investigated by determining theoretical relative peripheral power profiles experienced by a myopic eye requiring a −2.50 D distance prescription and perfectly accommodating for near target distances through the near zones of the 1.50 D PALs tested in this study and Schilling et al.’s study. The top two lines of Figure 8 show the sagittal and tangential components of power along the horizontal meridian for these lenses. The sagittal power profiles are similar, but there is a steeper decrease in tangential power away from the center in the Schilling's PAL 1 design than in our PAL 1 design (middle line). This occurs also when comparing our PAL 2 design with Schilling at al.’s PAL 4 design (bottom line), but mostly on the nasal side. It is thus possible that the differences between the lags with PALs in the two studies might be caused by the differences in lens designs used.

### Contrast between the autorefractor and aberrometer

Comparisons of the accommodative lags measured with the Grand-Seiko autorefractor and the COAS aberrometer when looking through the near zone of two progressive lenses, that have different power gradients around the near reference point, show not only different magnitudes of the lags but even a consistent change in sign of these differences (compare the last rows of Tables 2 and 4). This casts doubt about the ability of autorefractors to accurately predict the relative clinical efficacy of complex lens designs to reduce accommodative lag. It is concerning that most of the published research on accommodation relied on autorefractors to measure accommodative responses. Tables 1 and 2 of the updated IMI Report on accommodation in myopia (Logan et al., 2021) reveal that only two out of 27 listed accommodation studies used aberrometers for this task.

### Possible implications for myopia control in children

Both multifocal spectacle and contact lenses have been designed as accommodative aids and show similar efficacy in controlling progression of myopia over the first 12 months (Varnas et al., 2021). In the BLINK Study, (Walline et al., 2020) found that 2.50 D addition multifocal contact lenses were more effective in slowing myopia progression in children than lenses with a lower 1.50 D addition. In the case of progressive spectacle lenses, addition powers higher than 2.00 D have not been used in published clinical trials. For moderate addition powers of 1.50 D to 2.00 D that are typically used, the treatment effect tends to wane after the first 12 months (Varnas et al., 2021; Walline et al., 2020). Contributing to this diminishing myopia control effect is the fact that 1.50 D PALs became relatively ineffective at controlling the lag after this time, except for the largest target distance (40 cm).

When wearing progressive spectacle lenses, the accommodative lag increased as distance decreased. Consequently, for lenses to be effective in reducing accommodative lag, add power needs to be tailored to the habitual distances. The 1.50 D in a PAL can do this up to stimuli of ∼3 D but some children experience average stimuli of close to 5 D when using small electronic devices (Xu et al., 2020). If such devices dominate a child's near vision tasks, a higher addition power needs to be considered for the myopia control treatment from the start.

We found evidence that the accommodative lags in myopic adult subjects increase over time while wearing PALs. To maintain the treatment effect, a change in the intervention in the form of the boost to the addition power of the progressive lens may be needed. Our results suggest that a 0.50 D boost to the addition power after 12 months of wear could be beneficial to maintain sufficiently low accommodative lags across the range of stimuli examined. A future clinical study would be required to determine whether increasing the PAL add power at yearly intervals extends the efficacy of myopia control over more years.

The range of addition powers used in PALs for presbyopes is between 0.5 D and 4.0 D. We assume that a similar range could be used in PALs for children, although the shorter corridor lengths employed in children's PALs will make adaptation to very high adds challenging. We expect that the maximum addition power used for these lenses would not exceed 3.00 D, but the children should start with as low an add as is the minimum to give a tangible clinical effect (e.g., 25% or higher average retardation of progression). We recommend starting with at least 1.00 D add for children having no parental myopia and at least 1.50 D when both parents are myopic. This recommendation is based on the subgroup analysis in the Hasebe, Jun, and Varnas (2014) clinical trial, which has shown the lower addition power effective in reducing progression of myopia only in the subgroup of children with no parental myopia. Higher addition starting values may need to be considered, if the child tends to use very close object distances.

This study was based on the premise that the sustained accommodative lag during close work is a plausible cause of the onset and progression of myopia. Pärssinen & Kauppinen (2022) have found an association between close work time and incidence of myopia in a historical survey of around 5000 Finnish children. An association between longer near work sessions and incidence of myopia has been also been recently found in around 1400 children aged between three and 18 years old (Philipp et al., 2022). Close work is associated with the fovea and perifovea experiencing accommodative lag, not the peripheral retina, which has mostly accommodative lead during close work (Flitcroft, 2012). The peripheral retina experiences astigmatic errors, which means that it may have conflicting cues for eye growth. Figure 8 shows that, except for the SVL at 33 and 25 cm (bottom line), tangential power yields a hyperopic defocus relative to central vision, whereas the sagittal power creates a myopic defocus in the periphery relative to central vision. Given that animal experiments suggest myopic defocus to be more powerful than hyperopic defocus in regulating eye growth (Tse et al., 2007), it is unlikely that the peripheral retina provides an effective stimulus to eye growth during near vision when wearing an SVL or a PAL. That makes the hyperopic defocus on the fovea due to accommodative lag a more likely candidate for the stimulation of eye growth.

### Limitations

An obvious limitation of the study is the Covid-related dropout rate and the different cohorts available at different follow-up times; this has been already mentioned in the methods section. A second limitation is monocular stimulation of accommodation during measurements which may have led to higher accommodative lags than would be the case during binocular stimulation; however, it is unlikely that this would be important because the study concentrated on the differences between accommodative lags when wearing different lenses and how these changed over time.

The participants would have lost a small amount of accommodation ability over the 12 months of the study, but it is very unlikely that this affected the accommodation lag. León, Rosenfield, Estrada, Medrano, and Márquez (2017) found that lag of accommodation for a 2.5 D stimulus was constant at approximately 0.5 D between the ages of five and 39 years. With a 1.50 D addition, this corresponds to the highest stimulus for which the 18- to 27-year group was tested.

## Conclusions

PALs reduced accommodative lags compared with single-vision distance lenses. Effects varied according to the PAL design, measurement method and near target distance. After 12 months of wear, the accommodative effect of PALs diminished, but could be restored with 0.50 D and 0.75 D booster adds. Our recommendations are that for PALs to reduce accommodative lag effectively, addition power should be tailored to typical working distances, and after the first year of wear should be boosted by at least 0.50 D to maintain efficacy. These findings should be valid for all positive lens addition accommodative aids, including progressive and bifocal spectacle lenses, and bifocal and multifocal contact lenses.

## References

[bib1] Bao, J., Huang, Y., Li, X., Yang, A., Zhou, F., Wu, J., & Chen, H. (2022). Spectacle lenses with aspherical lenslets for myopia control vs single-vision spectacle lenses: A randomized clinical trial. *JAMA Ophthalmology**,* 140, 472–478. 35357402 10.1001/jamaophthalmol.2022.0401 PMC8972151

[bib2] Chamberlain, P., Peixoto-de-Matos, S. C., Logan, N. S., Ngo, C., Jones, D., & Young, G. (2019). A 3-year randomized clinical trial of MiSight lenses for myopia control. *Optometry and Vision Science**,* 96(8), 556–567. 31343513 10.1097/OPX.0000000000001410

[bib3] Flitcroft, D. I. (2012). The complex interactions of retinal, optical and environmental factors in myopia aetiology. *Progress in Retinal Eye Research**,* 31(6), 622–660. 22772022 10.1016/j.preteyeres.2012.06.004

[bib4] Hasebe, S., Jun, J., & Varnas, S. R. (2014). Myopia control with positively aspherized progressive addition lenses: a 2-year, multicenter, randomized, controlled trial. *Investigative Ophthalmology and Vision Science**,* 55(11), 7177–7188. 10.1167/iovs.12-11462 25270192

[bib5] Holden, B. A., Fricke, T. R., Wilson, D. A., Jong, M., Naidoo, K. S., Sankaridurg, P., & Resnikoff, S. (2016). Global prevalence of myopia and high myopia and temporal trends from 2000 through 2050. *Ophthalmology**,* 123(5), 1036–1042. 26875007 10.1016/j.ophtha.2016.01.006

[bib6] Kaphle, D., Varnas, S. R., Schmid, K. L., Suheimat, M., Leube, A., & Atchison, D. A. (2022). Accommodation lags are higher in myopia than emmetropia: measurement methods and metrics matter. *Ophthalmic and Physiological Optics**,* 42(6), 1103–1114. 35775299 10.1111/opo.13021 PMC9544228

[bib7] Labhishetty, V., Cholewiak, S. A., & Banks, M. S. (2019). Contributions of foveal and non-foveal retina to the human eye's focusing response. *Journal of Vision**,* 19(12):18, 11–25. 10.1167/19.12.18 31627211

[bib8] Labhishetty, V., Cholewiak, S. A., Roorda, A., & Banks, M. S. (2021). Lags and leads of accommodation in humans: Fact or fiction? *Journal of Vision**,* 21(3), 21. 10.1167/jov.21.3.21 PMC7995353 33764384

[bib9] Lam, C. S. Y., Tang, W. C., Tse, D. Y., Lee, R. P. K., Chun, R. K. M., Hasegawa, K., & To, C. H. (2020). Defocus Incorporated Multiple Segments (DIMS) spectacle lenses slow myopia progression: a 2-year randomised clinical trial. *British Journal of Ophthalmology**,* 104(3), 363–368. 31142465 10.1136/bjophthalmol-2018-313739 PMC7041503

[bib10] León, A., Rosenfield, M., Estrada, J. M., Medrano, S. M., & Márquez, M. M. (2017). Lag of accommodation between 5 and 60 years of age. *Optometry and Vision Performance**,* 5(3), 103–108.

[bib11] Logan, N. S., Radhakrishnan, H., Cruickshank, F. E., Allen, P. M., Bandela, P. K., Davies, L. N., & Wolffsohn, J. S. (2021). IMI accommodation and binocular vision in myopia development and progression. *Investigative Ophthalmology and Vision Science**,* 62(5):4, 1–21. 10.1167/iovs.62.5.4 PMC8083074 33909034

[bib12] Mathur, A., & Atchison, D. A. (2013). Peripheral refraction patterns out to large field angles. *Optometry and Vision Science**,* 90(2), 140–147. 23353991 10.1097/OPX.0b013e31827f1583

[bib13] Morgan, I. G., French, A. N., Ashby, R. S., Guo, X., Ding, X., He, M., et al. (2018). The epidemics of myopia: Aetiology and prevention. *Progress in Retinal Eye Research**,* 62, 134–149. 28951126 10.1016/j.preteyeres.2017.09.004

[bib14] Pärssinen, O., & Kauppinen, M. (2022). Associations of near work time, watching TV, outdoors time, and parents' myopia with myopia among school children based on 38-year-old historical data. *Acta Ophthalmologica**,* 100(2), e430–e438. 34291573 10.1111/aos.14980

[bib15] Philipp, D., Vogel, M., Brandt, M., Rauscher, G. G., Hiemisch, A., Wahl, S., & Poulain, T. (2022). The relationship between myopia and near work, time outdoors and socioeconomic status in children and adolescents. *BMC Public Health**,* 22:2058, 1–10. 36357862 10.1186/s12889-022-14377-1 PMC9650855

[bib16] Schilling, T., Ohlendorf, A., Varnas, S. R., & Wahl, S. (2017). Peripheral design of progressive addition lenses and the lag of accommodation in myopes. *Investigative Ophthalmology and Vision Science**,* 58(9), 3319–3324. 10.1167/iovs.17-21589 28672398

[bib17] Seijas, O., Gómez de Liaño, P., Gómez de Liaño, R., Roberts, C. J., Piedrahita, E., & Diaz, E. (2007). Ocular dominance diagnosis and its influence in monovision. *American Journal of Ophthalmology**,* 144, 209–216. 17533108 10.1016/j.ajo.2007.03.053

[bib18] Thibos, L. N., Hong, X., Bradley, A., & Applegate, R. A. (2004). Accuracy and precision of objective refraction from wavefront aberrations. *Journal of Vision**,* 4(4), 329–351. 15134480 10.1167/4.4.9

[bib19] Thibos, L. N., Wheeler, W., & Horner, D. (1997). Power vectors: An application of Fourier analysis to the description and statistical analysis of refractive error. *Optometry and Vision Science**,* 74(6), 367–375. 9255814 10.1097/00006324-199706000-00019

[bib20] Tse, D. Y., Lam, C. S., Guggenheim, J. A., Lam, C., Li, K. K., Liu, Q., et al. (2007). Simultaneous defocus integration during refractive development. *Investigative Ophthalmology and Vision Science**,* 48(12), 5352–5359. 10.1167/iovs.07-0383 18055781

[bib21] Varnas, S., Gu, X., & Metcalfe, A. (2021). Bayesian meta-analysis of myopia control with multifocal lenses. *Journal of Clinical Medicine**,* 10(4), 730. 33673218 10.3390/jcm10040730 PMC7917905

[bib22] Walline, J. J., Lindsley, K. B., Vedula, S. S., Cotter, S. A., Mutti, D. O., Ng, S. M., et al. (2020). Interventions to slow progression of myopia in children. *Cochrane Database Systematic Reviews**,* 1(1), Cd004916. 10.1002/14651858.CD004916.pub4 PMC6984636 31930781

[bib23] Walline, J. J., Walker, M. K., Mutti, D. O., Jones-Jordan, L. A., Sinnott, L. T., Giannoni, A. G., & Berntsen, D. A. (2020). Effect of high add power, medium add power, or single-vision contact lenses on myopia progression in children: The BLINK randomized clinical trial. *JAMA Ophthalmology**,* 324(6), 571–580. 10.1001/jama.2020.10834 PMC7420158 32780139

[bib24] Xu, R., Jaskulski, M., Bradley, A., Kollbaum, P. S., & Krueger, R. R. (2020). Viewing behavior of children using mobile phones. *Investigative Ophthalmology and Vision Science**,* 61(7), 1924.

[bib25] Yam, J. C., Jiang, Y., Tang, S. M., Law, A. K. P., Chan, J. J., Wong, E., & Pang, C. P. (2019). Low-concentration Atropine for Myopia Progression (LAMP) Study: A randomized, double-blinded, placebo-controlled trial of 0.05%, 0.025%, and 0.01% atropine eye drops in myopia control. *Ophthalmology**,* 126(1), 113–124. 30514630 10.1016/j.ophtha.2018.05.029

